# Supplementary material for: Evaluating multiple candidates simultaneously reduces racial disparities in promotion and tenure
Source: Nat Commun. 2026 Feb 23;17:3080. doi: 10.1038/s41467-026-69937-5 (PMC13039406; doi:10.1038/s41467-026-69937-5)
Supplement: Supplementary file 1 — Supplementary Information [file 41467_2026_69937_MOESM1_ESM.pdf]

Supplementary Materials

Joint Evaluation Reduces Racial Disparities in Promotion: Evidence from a Natural Experiment.

The PDF file includes:

Tables S1 to S21

Table S1. Promotion and tenure timelines for each institution.

**Institution**	**External Review Letters (ERLs)**	**Department Vote**	**College Vote**	**Dean's Letter/Vote**
University A	Faculty members finalize their dossiers between June 1 - September 1	Department votes occurs between September 1 - 30	College P&T committee complete reviews and recommendations from September 15 - October 20	Dean conducts recommendations and votes from October 21 - December 2
University B	Candidates must submit their dossiers to their respective department heads by mid-August	Department committee chairs provide evaluation letters, including their vote by mid-September	The College-wide committee reviews, provides feedback, and votes by late November	Deans review letter is submitted between Early December - Early January
University C	ERLs are solicited early in the fall and are typically due by mid-October	Department reviews and votes occur mid to late October	College P&T committees vote typically occurs mid-November	Deans recommendations are due prior to the end of the fall semester (mid-December)
University D	ERLs must be submitted by late September/early October	Department submit recommendations by early October	College P&T committee submit recommendations by mid-November	Dean recommendation and notification to candidates are submitted by early December
University E	Promotion candidates submit their dossier by September 1	Department chairs forward dossier, with department recommendation letters to Dean by October 1	College P&T committee submits recommendation to Dean by November 1	Dean forwards dossier, with P&T committee and Dean recommendation letters, to Provost by January 30
University F	ERLs are submitted by reviewers by late August	Department P&T committee and chair complete reviews by late September	College P&T committees complete reviews between late October - early November	Dean completes candidates dossier and informs Faculty Affairs by December 1

Table S2. Means and standard deviations of Study 2 variables broken down by university

	University 1		University 2		University 3		University 4		University 5		University 6	
	M	SD	M	SD	M	SD	M	SD	M	SD	M	SD
1. Joint evaluation	0.51	0.50	0.52	0.50	0.44	0.50	0.43	0.50	0.55	0.50	0.47	0.50
2. URM status	0.11	0.31	0.10	0.29	0.08	0.27	0.07	0.25	0.09	0.29	0.65	0.48
3. Promotion rank	0.43	0.50	0.55	0.50	0.43	0.50	0.41	0.49	0.41	0.49	0.61	0.49
4. Woman	0.40	0.49	0.34	0.47	0.34	0.47	0.35	0.48	0.40	0.49	0.52	0.50
5. Tenure in rank	5.88	3.50	6.18	3.36	5.83	3.52	6.34	3.17	6.19	2.76	6.03	2.47
6. External grants	4.59	5.46	6.67	9.94	5.38	5.57	3.11	4.27	3.65	5.12	0.75	1.75
7. Dept. negative vote %	0.04	0.13	0.09	0.20	0.08	0.20	0.04	0.12	0.09	0.20	0.16	0.36
8. Dept. unanimous vote	0.87	0.34	0.73	0.44	0.73	0.45	0.86	0.34	0.76	0.43	0.83	0.38
9. Total department votes	6.82	5.91	10.99	9.48	13.41	11.11	9.69	4.60	7.69	5.97	1.74	1.66

Note. This table represents means and standard deviations of Study 2 variables broken down by university. Joint evaluation is coded 1 for joint evaluation and 0 for single evaluation. URM status is coded 1 for candidates who are underrepresented minorities (Black/African American or Hispanic) and 0 for candidates who are White/Caucasian or Asian/Asian American. Promotion rank is coded 1 for promotion to full and 0 for promotion to associate. Woman is coded 1 for women candidates and 0 for men candidates. Tenure in rank refers to the number of years a candidate has been in their present rank. External grants refers to the number of external grants awarded as principal investigator. Dept. negative vote percentage is calculated as the total number of no votes divided by the total number of votes cast for a candidate. Dept. unanimous vote is coded 1 for a unanimous vote and 0 for a non-unanimous vote. Total department votes is used as a proxy for department size.

Table S3. Count of missing data for each variable used in analyses for the natural experiment.

Variable	# Missing
Joint Evaluation	0
Woman	0
Total Department Votes	0
URM Status	58
External Grants	196
Department Negative Vote Percentage	209
H Index	573

Note. Joint evaluation is coded 1 for joint evaluation and 0 for single evaluation. Woman is coded 1 for women candidates and 0 for men candidates. Total department votes is used as a proxy for department size. URM status is coded 1 for candidates who are underrepresented minorities (Black/African American or Hispanic) and 0 for candidates who are White/Caucasian or Asian/Asian American. External grants refers to the number of external grants awarded as principal investigator. Department negative vote percentage is calculated as the total number of no votes divided by the total number of votes cast for a candidate.

Table S4. Simple effects of joint evaluation on department negative vote percentage at both levels of URM status.

Moderator Values	b	95% CI	p
URM (1)	-.09	-0.17, -0.01	.026
Non-URM (0)	.03	0.01, 0.06	.009

Note. We calculated the simple effects from the final OLS regression model (i.e., All Controls) from Table 5 in the main text.

Table S5. Logistic regression results for the interactive effect (steps 1 & 5) of URM status and joint evaluation on department unanimous vote.

	Department Unanimous Votes					
	Model 1			Model 5		
	No Controls			All Controls		
Variable	OR	95% CI	p	OR	95% CI	p
URM status	0.93	0.85, 1.02	.132	0.94	0.82, 1.07	.351
Joint evaluation	0.92	0.88, .96	<.001	0.95	0.90, 1.00	.052
URM x Joint eval.	1.08	0.94, 1.23	.289	1.14	0.95, 1.36	.171
Women				0.98	0.93, 1.04	.572
Promotion rank				0.88	0.83, 0.93	<.001
Tenure in rank				0.99	0.98, 1.00	.013
H-index				1.00	1.00, 1.00	.889
External grants				1.00	0.99, 1.00	.524
Total dept. votes				0.99	0.98, 0.99	<.001
N		1556			1027	
Nagelkerke R^2		.01			.14	

Note. Logistic regression was conducted. Base controls included candidate a) institution, b) discipline, c) gender, d) promotion rank, and e) years in current rank. Institution and CIP Code were also used as controls but were not presented in the table due to the large number of parameters. URM status is coded 1 for candidates who are underrepresented minorities (Black/African American or Hispanic) and 0 for candidates who are White/Caucasian or Asian/Asian American. Joint evaluation is coded 1 for joint evaluation and 0 for single evaluation. Woman is coded 1 for women candidates and 0 for men candidates. Promotion rank is coded 1 for promotion to full and 0 for promotion to associate. Tenure in rank refers to the number of years a candidate has been in their present rank. H-index refers to the candidate's h-index at the time of P&T. External grants refers to the number of external grants awarded as principal investigator. Total dept. votes is used as a proxy for department size. No adjustments were made for multiple comparisons.

Table S6. Generalized Linear Modeling analysis with logit-link for the interactive effect of URM status and joint evaluation (also termed Joint Eval.) on department vote outcome (binary: 1 "any negative vote", 0 "no negative votes")

Department Negative Vote (1 = any negative vote, 0 = unanimous)			
Variable	OR	95% CI	p
URM status	1.54	0.74, 3.21	.248
Joint evaluation	1.45	1.05, 2.00	.025
URM x Joint eval.	.39	0.13, 1.14	.084
Women	1.03	0.75, 1.43	.860
Promotion rank	2.25	1.57. 3.22	<.001
Tenure in rank	1.04	1.00, 1.08	.063
H-index	1.00	0.98, 1.01	.850
External grants	1.01	0.99,1.03	.174
Total department votes	1.07	1.05, 1.09	<.001
N		1027	
Nagelkerke R^2		.61	

Note. Institution and CIP Code were also used as controls but were not presented in the table due to the large number of parameters. URM status is coded 1 for candidates who are underrepresented minorities (Black/African American or Hispanic) and 0 for candidates who are White/Caucasian or Asian/Asian American. Joint evaluation is coded 1 for joint evaluation and 0 for single evaluation. Woman is coded 1 for women candidates and 0 for men candidates. Promotion rank is coded 1 for promotion to full and 0 for promotion to associate. Tenure in rank refers to the number of years a candidate has been in their present rank. H-index refers to the candidate's h-index at the time of P&T. External grants refers to the number of external grants awarded as principal investigator. Total department votes is used as a proxy for department size.

Table S7. Moderated mediation results for conditional indirect effect of URM status and joint evaluation on Provost vote via department negative vote percentage.

	Department Negative Vote %			Provost vote		
Variable	b	95% CI	p	b	95% CI	p
URM status	.08	.02, .14	.012	0.66	-0.76, 2.09	.362
Joint evaluation	.04	.01, .06	.007	-5.00	-6.14, -3.86	<.001
URM x Joint eval.	-.13	-.21, -.04	.004			
Women	-.01	-.04, .01	.374	-.26	-1.13, .61	.563
Promotion rank	.03	.005, .06	.021	.38	-.57, 1.33	.428
Tenure in rank	.01	.002, .01	.001	-.12	-.19, -.04	.002
H-index	-.0002	-.002, .001	.699	.001	-.04, .04	.957
External grants	-.00	-.002, .002	.992	-.005	-.06, .05	.837
Total dept. votes	-.003	-.002, .0001	.726	.02	-.04, .09	.471
Dept. Negative Vote %				-4.88	-6.05, -3.70	<.001
N		909			909	
R^2		.06			.39	

	Effect	bootCI	p
Separate evaluation	-.39	-.92, .05	.113
Joint evaluation	.23	.03, .46	.017
IMM	.61	.15, 1.21	.025

Note. Institution and CIP Code were also used as controls but were not presented in the table due to the large number of parameters. URM status is coded 1 for candidates who are underrepresented minorities (Black/African American or Hispanic) and 0 for candidates who are White/Caucasian or Asian/Asian American. Joint evaluation is coded 1 for joint evaluation and 0 for single evaluation. Woman is coded 1 for women candidates and 0 for men candidates. Promotion rank is coded 1 for promotion to full and 0 for promotion to associate. Tenure in rank refers to the number of years a candidate has been in their present rank. H-index refers to the candidate's h-index at the time of P&T. External grants refers to the number of external grants awarded as principal investigator. Total dept. votes is used as a proxy for department size. IMM refers to Index of Moderated Mediation.

Table S8. OLS regression models for the interactive effect of URM status and joint evaluation (also termed Joint Eval.) on department negative vote percentage using clustered standard errors for universities (Model 1) and departments (Model 2).

	Department Negative Vote Percentage					
	Cluster SE on Universities			Cluster SE on Departments		
Variable	b	95% CI	p	b	95% CI	p
URM status	.07	-.02, .04	.129	.07	-.02, .16	.124
Joint evaluation	.04	.03, .04	<.001	.04	.009, .06	.009
URM x Joint eval.	-.13	-.21, -.05	.002	-.13	-.22, -.03	.009
Women	-.01	-.02, .01	.454	-.01	-.03, .02	.645
Promotion rank	.03	.02, .05	<.001	.03	.01, .06	.008
Tenure in rank	.01	.003, .01	<.001	.01	0.01, .01	.024
H-index	.00	-.001, .001	.993	.00	-.002, .002	.996
External grants	-.004	-.001, .0001	.118	-.004	-.002, .001	.574
Total dept. votes	-.01	-.001, .0002	.144	-.01	-.001, .0004	.283
N		1027			1027	
R^2		.08			.08	

Note. Institution and CIP Code were also used as controls but were not presented in the table due to the large number of parameters. URM status is coded 1 for candidates who are underrepresented minorities (Black/African American or Hispanic) and 0 for candidates who are White/Caucasian or Asian/Asian American. Joint evaluation is coded 1 for joint evaluation and 0 for single evaluation. Woman is coded 1 for women candidates and 0 for men candidates. Promotion rank is coded 1 for promotion to full and 0 for promotion to associate. Tenure in rank refers to the number of years a candidate has been in their present rank. H-index refers to the candidate's h-index at the time of P&T. External grants refers to the number of external grants awarded as principal investigator. Total department votes is used as a proxy for department size.

Table S9. OLS regression model for the interactive effect of URM status and joint evaluation (also termed Joint Eval.) on department negative vote percentage using university-by-CIP fixed effects

	Department Negative Vote %		
Variable	b	95% CI	p
URM status	.06	-.04, .16	.195
Joint evaluation	.02	.01, .04	.015
URM x Joint eval.	-.12	-.23, -.01	.037
Women	.0004	-.01, .01	.931
Promotion rank	.04	.03, .06	.001
Tenure in rank	.01	.002, .01	.016
H-index	-.0002	-.002, .001	.774
External grants	-.001	-.002, -.00003	.045
Total dept. votes	.001	-.0003, .002	.152
N		1027	
R^2		.04	

Note. Institution and CIP Code were also used as controls but were not presented in the table due to the large number of parameters. URM status is coded 1 for candidates who are underrepresented minorities (Black/African American or Hispanic) and 0 for candidates who are White/Caucasian or Asian/Asian American. Joint evaluation is coded 1 for joint evaluation and 0 for single evaluation. Woman is coded 1 for women candidates and 0 for men candidates. Promotion rank is coded 1 for promotion to full and 0 for promotion to associate. Tenure in rank refers to the number of years a candidate has been in their present rank. H-index refers to the candidate's h-index at the time of P&T. External grants refers to the number of external grants awarded as principal investigator. Total department votes is used as a proxy for department size.

Table S10. Stepwise OLS regression models for the interactive effect of URM status and joint evaluation (also termed Joint Eval.) on department negative vote percentage (Native Americans included as URMs).

	Department Negative Vote %														
	Model 1			Model 2			Model 3			Model 4			Model 5		
	No Controls			Base Controls			Productivity			Department Size			All Controls		
Variable	b	95% CI	p	b	95% CI	p	b	95% CI	p	b	95% CI	p	b	95% CI	p
URM status	.10	0.05, 0.14	<.001	.08	0.04, 0.13	<.001	.07	0.01, 0.13	.029	.08	0.04, 0.13	<.001	.07	0.01, 0.13	.028
Joint evaluation	.04	0.01, 0.06	.001	.04	0.02, 0.06	.001	.03	0.01, 0.06	.010	.04	0.02, 0.06	.001	.03	0.01, 0.06	.01
URM x Joint eval.	-.09	-0.16, -0.03	.004	-.09	-0.16, -0.02	.007	-.12	-0.21, -0.04	.004	-.09	-0.16, -0.02	.007	-.12	-0.21, -0.04	.004
Women				-.004	-0.03, 0.02	.700	-.01	-0.03, 0.02	.717	-.004	-0.03, 0.02	.699	-.01	-0.03, 0.02	.726
Promotion rank				.02	0.00, 0.04	.052	.04	0.01, 0.07	.006	.02	0.00, 0.04	.072	.04	0.01, 0.06	.016
Tenure in rank				.01	0.00, 0.01	.001	.01	0.00, 0.01	.001	.01	0.00, 0.01	.001	.01	0.00, 0.01	.001
H-index							.00	-0.01, 0.01	.924				.001	-0.01, 0.02	.865
External grants							-.001	-0.00, 0.00	.295				-.001	-0.00, 0.00	.297
Total dept. votes										-.00	-0.00, 0.00	.886	-.00	-0.00, 0.00	.624
N		1557			1537			1028			1537			1028	
R^2		.02			.07			.08			.07			.08	

Note. Base controls included candidate a) institution, b) discipline, c) gender, d) promotion rank, and e) years in current rank. Institution and CIP Code were also used as controls in Models 2, 3, 4, and 5 but were not presented in the table due to the large number of parameters. URM status is coded 1 for candidates who are underrepresented minorities (Black/African American, Hispanic, or Native American) and 0 for candidates who are White/Caucasian or Asian/Asian American. Joint evaluation is coded 1 for joint evaluation and 0 for single evaluation. Woman is coded 1 for women candidates and 0 for men candidates. Promotion rank is coded 1 for promotion to full and 0 for promotion to associate. Tenure in rank refers to the number of years a candidate has been in their present rank. H-index refers to the candidate's h-index at the time of P&T. External grants refers to the number of external grants awarded as principal investigator. Total dept. votes is used as a proxy for department size. No adjustments were made for multiple comparisons.

Table S11. Interactive effect of URM status and joint evaluation (also termed Joint Eval.) on department negative vote percentage with missing value dummy variables.

	Department Negative Vote %		
Variable	b	95% CI	p
URM status	.09	.05, .14	<.001
Joint evaluation	.04	.01, .06	<.001
URM x Joint eval.	-.10	-.16, -.03	.003
Women	-.01	-.03, .01	.513
Promotion rank	.02	-.001, .04	.065
Tenure in rank	.004	.001, .01	.011
H-index	.0004	-.001, .002	.532
H-index_missing	.01	-.02, .04	.359
External grants	-.0003	-.002, .001	.687
External grants_missing	.01	-.03, .04	.729
Total dept. votes	-.001	-.002, .0003	.158
N		1535	
R^2		.04	

Note. Institution and CIP Code were also used as controls but were not presented in the table due to the large number of parameters. URM status is coded 1 for candidates who are underrepresented minorities (Black/African American or Hispanic) and 0 for candidates who are White/Caucasian or Asian/Asian American. Joint evaluation is coded 1 for joint evaluation and 0 for single evaluation. Woman is coded 1 for women candidates and 0 for men candidates. Promotion rank is coded 1 for promotion to full and 0 for promotion to associate. Tenure in rank refers to the number of years a candidate has been in their present rank. H-index refers to the

candidate's h-index at the time of P&T. External grants refers to the number of external grants awarded as principal investigator. Total department votes is used as a proxy for department size.

Table S12. Bootstrapped OLS regression results for the effect of joint evaluation on department negative vote percentage with 5,000 iterations.

	Department Negative Vote %		
Variable	b	95% CI	p
URM status	.07	0.01, 0.13	.025
Joint evaluation	.03	0.01, 0.06	.006
URM x Joint eval.	-.12	-0.20, -0.04	.004
Women	-.01	-0.03, 0.02	.650
Promotion rank	.04	0.01, 0.06	.011
Tenure in rank	.01	0.003, 0.01	<.001
H-index	.00	-0.01, 0.01	.995
External grants	-.001	-0.002, 0.001	566
Total dept. votes	.001	-0.002, 0.001	.476
N		1027	
R^2		.08	

Note. Institution and CIP Code were also used as controls but were not presented in the table due to the large number of parameters. URM status is coded 1 for candidates who are underrepresented minorities (Black/African American or Hispanic) and 0 for candidates who are White/Caucasian or Asian/Asian American. Joint evaluation is coded 1 for joint evaluation and 0 for single evaluation. Woman is coded 1 for women candidates and 0 for men candidates. Promotion rank is coded 1 for promotion to full and 0 for promotion to associate. Tenure in rank refers to the number of years a candidate has been in their present rank. H-index refers to the candidate's h-index at the time of P&T. External grants refers to the number of external grants awarded as principal investigator. Total department votes is used as a proxy for department size.

Table S13. OLS regression results for the interactive effect of URM status and joint evaluation on department negative vote percentage excluding candidates who received zero negative votes.

	Department Negative Vote %		
Variable	b	95% CI	p
URM status	.17	0.02, 0.32	.027
Joint evaluation	.03	-0.04, 0.10	.355
URM x Joint eval.	-.30	-.52, -.07	.011
Women	-.04	-.11, .03	.268
Promotion rank	.001	-.07, .07	.978
Tenure in rank	.01	.001, .02	.022
H-index	-.001	-.004, .002	.399
External grants	-.005	-.01, -.001	.020
Total dept. votes	-.01	-.01, -.01	<.001
N		375	
R^2		.27	

Note. Institution and CIP Code were also used as controls but were not presented in the table due to the large number of parameters. URM status is coded 1 for candidates who are underrepresented minorities (Black/African American or Hispanic) and 0 for candidates who are White/Caucasian or Asian/Asian American. Joint evaluation is coded 1 for joint evaluation and 0 for single evaluation. Woman is coded 1 for women candidates and 0 for men candidates. Promotion rank is coded 1 for promotion to full and 0 for promotion to associate. Tenure in rank refers to the number of years a candidate has been in their present rank. H-index refers to the candidate's h-index at the time of P&T. External grants refers to the number of external grants awarded as principal investigator. Total department votes is used as a proxy for department size.

Table S14. Stepwise random effect models for the interactive effect of URM status and joint evaluation (also termed Joint Eval.) on department negative vote percentage.

	Department Negative Vote %														
	Model 1			Model 2			Model 3			Model 4			Model 5		
	No Controls			Base Controls			Productivity			Department Size			All Controls		
Variable	b	95% CI	p	b	95% CI	p	b	95% CI	p	b	95% CI	p	b	95% CI	p
URM status	.10	0.06, 0.14	<.001	.09	0.05, 0.14	<.001	.07	0.01, 0.13	.022	.09	0.05, 0.14	<.001	.07	0.01, 0.13	.022
Joint evaluation	.03	0.01, 0.05	.005	.03	0.01, 0.05	.006	.03	0.01, 0.06	.009	.03	0.01, 0.05	.006	.03	0.01, 0.06	.009
URM x Joint eval.	-.09	-0.16, -0.03	.003	-.10	-0.16, -0.03	.002	-.12	-0.21, -0.04	.003	-.10	-0.16, -0.02	.003	-.12	-0.21, -0.04	.003
Women				-.001	-0.03, 0.02	.635	-.004	-0.03, 0.02	.752	-.005	-0.03, 0.02	.631	-.00	-0.03, 0.02	.750
Promotion rank				.02	-0.00, 0.04	.062	.04	0.01, 0.06	.006	.02	-0.00, 0.04	.109	.04	0.01, 0.06	.011
Tenure in rank				.04	0.00, 0.01	.006	.01	0.00, 0.01	.002	.04	0.00, 0.01	.006	.01	0.00, 0.01	.002
H-index							-.001	-0.01, 0.01	.897				-.00	-0.01, 0.01	.921
External grants							-.001	-0.00, 0.00	.379				-.00	-0.00, 0.00	.377
Total dept. votes										-.00	-0.00, 0.00	.598	-.00	-0.00, 0.00	.711
N		1556			1535			1027			1535			1027	
R^2		.02			.03			.04			.03			.04	

Note. Base controls included candidate a) institution, b) discipline, c) gender, d) promotion rank, and e) years in current rank. Institution and CIP Code were also used as controls in Models 2, 3, 4, and 5 but were not presented in the table due to the large number of parameters. URM status is coded 1 for candidates who are underrepresented minorities (Black/African American, Hispanic, or Native American) and 0 for candidates who are White/Caucasian or Asian/Asian American. Joint evaluation is coded 1 for joint evaluation and 0 for single evaluation. Woman is coded 1 for women candidates and 0 for men candidates. Promotion rank is coded 1 for promotion to full and 0 for promotion to associate. Tenure in rank refers to the number of years a candidate has been in their present rank. H-index refers to the candidate's h-index at the time of P&T. External grants refers to the number of external grants awarded as principal investigator. Total dept. votes is used as a proxy for department size. No adjustments were made for multiple comparisons.

Table S15. OLS regression results for the interactive effect of URM status and joint evaluation on department negative vote percentage for candidates seeking promotion to associate professor.

	Department Negative Vote %		
Variable	b	95% CI	p
URM status	.10	0.00, 0.20	.055
Joint evaluation	.01	-0.02, 0.05	.346
URM x Joint evaluation	-.13	-0.25, -0.01	.040
Women	-.01	-0.04, 0.02	.678
Tenure in rank	.03	0.02, 0.04	<.001
H-index	-.00	-0.02, 0.02	.775
External grants	-.003	-0.01, 0.00	.123
Total department votes	-.00	-0.00, 0.00	.841
R^2		.11	

Note. $N = 533$. Institution and CIP Code were also used as controls but were not presented in the table due to the large number of parameters. URM status is coded 1 for candidates who are underrepresented minorities (Black/African American or Hispanic) and 0 for candidates who are White/Caucasian or Asian/Asian American. Joint evaluation is coded 1 for joint evaluation and 0 for single evaluation. Woman is coded 1 for women candidates and 0 for men candidates. Tenure in rank refers to the number of years a candidate has been in their present rank. H-index refers to the candidate's h-index at the time of P&T. External grants refers to the number of external grants awarded as principal investigator. Total department votes is used as a proxy for department size. No adjustments were made for multiple comparisons.

Table S16. OLS regression results for the interactive effect of URM status and joint evaluation on department negative vote percentage for candidates seeking promotion to full professor.

	Department Negative Vote %		
Variable	b	95% CI	p
URM status	.07	-0.02, 0.16	.132
Joint evaluation	.06	0.02, 0.10	.007
URM x Joint evaluation	-.15	-0.28, -0.02	.025
Women	-.01	-0.05, 0.04	.744
Tenure in rank	.004	0.00, 0.01	.078
H-index	.001	-0.01, 0.03	.490
External grants	.00	-0.00, 0.00	.926
Total department votes	-.002	-0.01, 0.00	.305
R^2		.12	

Note. N = 494. Institution and CIP Code were also used as controls but were not presented in the table due to the large number of parameters. URM status is coded 1 for candidates who are underrepresented minorities (Black/African American or Hispanic) and 0 for candidates who are White/Caucasian or Asian/Asian American. Joint evaluation is coded 1 for joint evaluation and 0 for single evaluation. Woman is coded 1 for women candidates and 0 for men candidates. Tenure in rank refers to the number of years a candidate has been in their present rank. H-index refers to the candidate's h-index at the time of P&T. External grants refers to the number of external grants awarded as principal investigator. Total department votes is used as a proxy for department size. No adjustments were made for multiple comparisons.

Table S17. OLS regression results for the interactive effect of URM status and joint evaluation on department negative vote percentage (excluding cases where URMs are evaluated jointly with only other URMs)

Variable	Department Negative Vote %		
	b	95% CI	p
URM status	.07	0.01, 0.04	.025
Joint evaluation	.03	0.01, 0.06	.009
URM x Joint evaluation	-.12	-0.21, -0.04	.006
Women	-.01	-0.03, 0.02	.741
Promotion in rank	.04	0.01, 0.06	.018
Tenure in rank	.01	0.00, 0.01	.001
H-index	.00	-0.01, 0.02	.883
External grants	-.001	-0.00, 0.00	.305
Total department votes	-.00	-0.00, 0.00	.622
R^2		.08	

Note. $N = 1018$. Institution and CIP Code were also used as controls but were not presented in the table due to the large number of parameters. URM status is coded 1 for candidates who are underrepresented minorities (Black/African American or Hispanic) and 0 for candidates who are White/Caucasian or Asian/Asian American. Joint evaluation is coded 1 for joint evaluation and 0 for single evaluation. Woman is coded 1 for women candidates and 0 for men candidates. Promotion rank is coded 1 for promotion to full and 0 for promotion to associate. Tenure in rank refers to the number of years a candidate has been in their present rank. H-index refers to the candidate's h-index at the time of P&T. External grants refers to the number of external grants awarded as principal investigator. Total department votes is used as a proxy for department size. No adjustments were made for multiple comparisons.

Table S18. OLS regression results for the effect of joint evaluation on department negative vote percentage (excluding cases where URMs are evaluated alongside non-URMs)

Variable	Department Negative Vote %		
	b	95% CI	p
URM status	.08	0.01, 0.14	.017
Joint evaluation	.03	0.00, 0.06	.026
URM x Joint eval.	-.14	-0.29, 0.01	.069
Women	-.02	-0.05, 0.01	.189
Promotion in rank	.04	0.01, 0.07	.020
Tenure in rank	.01	0.00, 0.01	.007
H-index	.00	-0.01, 0.02	.805
External grants	-.001	-0.00, 0.00	.266
Total department votes	-.00	-0.00, 0.00	.619
R^2		.08	

Note. N = 941. Institution and CIP Code were also used as controls but were not presented in the table due to the large number of parameters. URM status is coded 1 for candidates who are underrepresented minorities (Black/African American or Hispanic) and 0 for candidates who are White/Caucasian or Asian/Asian American. Joint evaluation is coded 1 for joint evaluation and 0 for single evaluation. Woman is coded 1 for women candidates and 0 for men candidates. Promotion rank is coded 1 for promotion to full and 0 for promotion to associate. Tenure in rank refers to the number of years a candidate has been in their present rank. H-index refers to the candidate's h-index at the time of P&T. External grants refers to the number of external grants awarded as principal investigator. Total department votes is used as a proxy for department size. No adjustments were made for multiple comparisons.

Table S19. OLS regression results for the effect of candidate gender and joint evaluation on department negative vote percentage

Variable	Department Negative Vote %					
	No Interaction			All Controls		
	b	95% CI	p	b	95% CI	p
Women	-.01	-0.03, 0.01	.456	-.02	-0.06, 0.02	.313
Joint evaluation	.01	0.00, 0.04	.015	.01	-0.02, 0.04	.345
Women x Joint eval.				.03	-0.02, 0.08	.284
URM status				.003	-0.04, 0.05	.889
Promotion in rank				.04	0.01, 0.07	.011
Tenure in rank				.01	0.00, 0.01	<.001
H-index				.001	-0.01, 0.02	.853
External grants				-.001	-0.00, 0.00	.295
Total dept. votes				-.001	-0.00, 0.00	.555
N		1595			1027	
R^2		.004			.07	

Note. Institution and CIP Code were also used as controls but were not presented in the table due to the large number of parameters. Joint evaluation is coded 1 for joint evaluation and 0 for single evaluation. URM status is coded 1 for candidates who are underrepresented minorities (Black/African American or Hispanic) and 0 for candidates who are White/Caucasian or Asian/Asian American. Promotion rank is coded 1 for promotion to full and 0 for promotion to associate. Tenure in rank refers to the number of years a candidate has been in their present rank. H-index refers to the candidate's h-index at the time of P&T. External grants refers to the number of external grants awarded as principal investigator. Total depart. votes is used as a proxy for department size. No adjustments were made for multiple comparisons.

Table S20. Logistic regression results for the interactive effect of candidate gender and joint evaluation on department unanimous vote

	Department Unanimous Vote					
	No Interaction			All Controls		
Variable	OR	95% CI	p	OR	95% CI	p
Women	1.02	0.98, 1.07	.292	0.99	0.92, 1.07	.828
Joint evaluation	0.93	0.89, 0.97	.001	0.96	0.90, 1.02	.230
Women x Joint eval.				0.99	0.88, 1.10	.793
URM status				1.01	0.92, 1.10	.910
Promotion in rank				0.88	0.82, 0.93	<.001
Tenure in rank				0.99	0.98, 1.00	.010
H-index				1.00	1.00, 1.00	.901
External grants				1.00	0.99, 1.00	.517
Total dept. votes				0.99	0.98, 0.99	<.001
N		1595			1027	
R^2		.01			.13	

Note. *N* = 941. Institution and CIP Code were also used as controls but were not presented in the table due to the large number of parameters. Joint evaluation is coded 1 for joint evaluation and 0 for single evaluation. URM status is coded 1 for candidates who are underrepresented minorities (Black/African American or Hispanic) and 0 for candidates who are White/Caucasian or Asian/Asian American. Promotion rank is coded 1 for promotion to full and 0 for promotion to associate. Tenure in rank refers to the number of years a candidate has been in their present rank. H-index refers to the candidate's h-index at the time of P&T. External grants refers to the number of external grants awarded as principal investigator. Total depart. votes is used as a proxy for department size. No adjustments were made for multiple comparisons.

Table S21. Partitioned R for each variable in Table 5, model including all control variables.

Variable	Department Negative Vote %
	Partitioned R^2
URM status	.008
Joint evaluation	.008
URM x Joint evaluation	.02
Women	.00
Tenure in rank	.02
Promotion in rank	.03
H-index	.008
External grants	.008
Total department votes	.003

Note. Institution and CIP Code were also used as controls but were not presented in the table due to the large number of parameters. URM status is coded 1 for candidates who are underrepresented minorities (Black/African American or Hispanic) and 0 for candidates who are White/Caucasian or Asian/Asian American. Joint evaluation is coded 1 for joint evaluation and 0 for single evaluation. Woman is coded 1 for women candidates and 0 for men candidates. Promotion rank is coded 1 for promotion to full and 0 for promotion to associate. Tenure in rank refers to the number of years a candidate has been in their present rank. H-index refers to the candidate's h-index at the time of P&T. External grants refers to the number of external grants awarded as principal investigator. Total department votes is used as a proxy for department size.
