# Supplementary material for: Evaluating multiple candidates simultaneously reduces racial disparities in promotion and tenure
Source: Nat Commun. 2026 Feb 23;17:3080. doi: 10.1038/s41467-026-69937-5 (PMC13039406; doi:10.1038/s41467-026-69937-5)
Supplement: Supplementary file 2 — Reporting Summary [file 41467_2026_69937_MOESM2_ESM.pdf]

nature portfolio

Corresponding author(s): Christiane Spitzmueller, Heyao Yu

Last updated by author(s): Jan 5, 2026

Reporting Summary

Nature Portfolio wishes to improve the reproducibility of the work that we publish. This form provides structure for consistency and transparency in reporting. For further information on Nature Portfolio policies, see our Editorial Policies and the Editorial Policy Checklist.

Statistics

For all statistical analyses, confirm that the following items are present in the figure legend, table legend, main text, or Methods section.

n/a	Confirmed	
☐	☒	The exact sample size (*n*) for each experimental group/condition, given as a discrete number and unit of measurement
☐	☒	A statement on whether measurements were taken from distinct samples or whether the same sample was measured repeatedly
☐	☒	The statistical test(s) used AND whether they are one- or two-sided<br>*Only common tests should be described solely by name; describe more complex techniques in the Methods section.*
☐	☒	A description of all covariates tested
☐	☒	A description of any assumptions or corrections, such as tests of normality and adjustment for multiple comparisons
☐	☒	A full description of the statistical parameters including central tendency (e.g. means) or other basic estimates (e.g. regression coefficient) AND variation (e.g. standard deviation) or associated estimates of uncertainty (e.g. confidence intervals)
☐	☒	For null hypothesis testing, the test statistic (e.g. *F*, *t*, *r*) with confidence intervals, effect sizes, degrees of freedom and *P* value noted<br>*Give P values as exact values whenever suitable.*
☒	☐	For Bayesian analysis, information on the choice of priors and Markov chain Monte Carlo settings
☒	☐	For hierarchical and complex designs, identification of the appropriate level for tests and full reporting of outcomes
☒	☐	Estimates of effect sizes (e.g. Cohen's *d*, Pearson's *r*), indicating how they were calculated

Our web collection on statistics for biologists contains articles on many of the points above.

Software and code

Policy information about availability of computer code

Data collection	No software was used.
Data analysis	Analyses were conducted using R, the code is provided on our OSF page (https://osf.io/jux2c/?view_only=fa50292557b5461aace147420501ad5b).

For manuscripts utilizing custom algorithms or software that are central to the research but not yet described in published literature, software must be made available to editors and reviewers. We strongly encourage code deposition in a community repository (e.g. GitHub). See the Nature Portfolio guidelines for submitting code & software for further information.

Data

Policy information about availability of data

All manuscripts must include a data availability statement. This statement should provide the following information, where applicable:

- Accession codes, unique identifiers, or web links for publicly available datasets
- A description of any restrictions on data availability
- For clinical datasets or third party data, please ensure that the statement adheres to our policy

Due to the extreme sensitivity of the data, variables that could potentially reveal the identity of the candidates or the institution were not made publicly available. Following this approach, one variable that was omitted from the publicly available data set is the US News ranking, because based on the ranking of the universities individuals would be able to identify the identity of the institutions, which we intend to keep private. All other study variables are publicly available and the partial subset of the data has been made publicly available at OSF (https://osf.io/jux2c/?view_only=fa50292557b5461aace147420501ad5b).

Research involving human participants, their data, or biological material

Policy information about studies with human participants or human data. See also policy information about sex, gender (identity/presentation), and sexual orientation and race, ethnicity and racism.

Reporting on sex and gender	This archival dataset included men and women who went up for promotion and tenure. The gender breakdown for candidates is 63% men and 37% women.
Reporting on race, ethnicity, or other socially relevant groupings	This paper examines racial disparities in promotion and tenure outcomes for historically underrepresented minorities. Using the definition from the National Science Foundation, historically underrepresented minorities within academia include Blacks or African Americans, Hispanics or Latinos, and American Indians or Alaska Natives. The ethnicity breakdown for candidates is 59% White, 25% Asian, 5% Hispanic, 7% Black, and 4% Other.
Population characteristics	The population examined in this study is university faculty seeking promotion and tenure across six universities. For the reviewers only, we can share the state location of our institutions: Texas (3), Pennsylvania (1), Alabama (1), and Louisiana (1).
Recruitment	Universities provided data on all promotion and tenure cases over a seven year period.
Ethics oversight	This study was reviewed by the primary institution's Institutional Review Board and declared to be non-human subjects research (STUDY00002463; MOD00003374). Each university's Academic Affairs leadership granted access to the archival data in accordance with the National Science Foundation policies. The IRB declaration from the primary university was then used to create IRB reliance agreements for the other partner institutions to agree to.

Note that full information on the approval of the study protocol must also be provided in the manuscript.

Field-specific reporting

Please select the one below that is the best fit for your research. If you are not sure, read the appropriate sections before making your selection.

☐ Life sciences ☒ Behavioural & social sciences ☐ Ecological, evolutionary & environmental sciences

For a reference copy of the document with all sections, see nature.com/documents/nr-reporting-summary-flat.pdf

Behavioural & social sciences study design

All studies must disclose on these points even when the disclosure is negative.

Study description	This was a quantitative study using 1) a survey of academic faculty (Study 1) and 2) archival data on promotion and tenure decisions from across 6 universities (Study 2).
Research sample	Study 1<br>This sample focused on faculty serving at US universities. The data was collected from across 11 universities in the United States. A total of 285 participants completed the survey, of which 77 were women, with the following race/ethnic breakdown: White (72.60%), Asian (11.74%), Hispanic (7.12%), Black/African American (3.91%), Native American, Native Alaskan, Pacific Islander (.71%), or Other (3.91%). Faculty members age was not recorded as this personal information was not deemed relevant to the current investigation.<br><br>Study 2<br>This was an archival dataset of promotion and tenure decisions made across six universities over a seven year period. The sample was faculty going up for promotion to associate or full professor within that time period. Most candidates were men (63%) and Caucasian/White (59%). Faculty members age was not recorded as this personal information was not deemed relevant to the current investigation.
Sampling strategy	Study 1<br>This was a convinience sample with no sample size calculation performed. The sample was not intended to be representative but instead obtain responses to the survey from individuals with experiences serving on P&T committees, this sample was chosen as they can answer the survey based on their personal experience.. The goal was to obtain a sample of roughly 300 faculty members. Data collection started on Feb. 19th and ended Feb 21st 2024.<br><br>Study 2<br>This study used an archival dataset of promotion and tenure decisions made across six universities. We included all data from universities on all promotion and tenure cases over a seven year period. Data collection was completed between 2015 and 2024.
Data collection	Study 1<br>Data was collected via an email sent to faculty via their institution email address. The survey was administered through Qualtrics<br><br>Study 2<br>Given the sensitivity of this data, a coding protocol was developed to ensure that faculty data would only be accessed locally and entered into individual spreadsheets per institution exclusively by individuals who worked in the provost's office or the equivalent and who typically had access to this confidential data prior to this project due to their regular job responsibilities. Personnel at each institution selected coders who already had access to promotion and tenure data and portfolios through their institutional role.

Institutional data included P&T processes, candidate characteristics (i.e., faculty demographics, discipline, tenure at the university, the rank they were seeking promotion to, and voting outcomes), external review letters (ERLs), writer characteristics, and ERL linguistic features. We held biweekly meetings with coders at each institution to clarify coding questions and ensure consistent coding practices across schools. Senior administrators who worked with our project cross-checked 2% of the cases for coding errors and refined coding practices in collaboration with the coders. No coders or administrators who cross-checked data were involved in the P&T decision-making processes at the respective institutions, nor did they attend P&T committee meetings or deliberations.

The research team merged the completed coding sheets that institution-based coders provided into the dataset. This dataset initially included an identifier for each candidate used to scrape productivity data (i.e., publication statistics and patents) from Google Scholar profiles and Academic Analytics. The scraped information is stored in a database separate from the dataset used in the analysis. The scraping identifier was imported into the database, assigning each candidate a (randomized) candidate number. The primary identifiable data was then removed from the dataset.

Timing

Archival data was collected over a seven year period (2015-2022) for six universities.

Data exclusions

Study 1
Only faculty who had served on P&T committees before were included in the data analyses.

Study 2
This data was collected by the Center for Excellence in Faculty Advancement (CEFA), a consortium of ten universities who collected data on P&T decisions over a seven year period. A subset of the archival data available was analyzed, with exclusion criteria being applied.

First, four universities were not able to provide the appropriate data (i.e., P&T voting outcomes) in order to be included in the analyses (N=241 excluded).

Non-participation

Study 1
Particiapnts were free to leave the study at any point.

Study 2
The data was archival.

Randomization

The data in Study 2 used quasi-random natural variation, and Study 1 had no randomization, so no experimenter randomization was used across the studies.

Reporting for specific materials, systems and methods

We require information from authors about some types of materials, experimental systems and methods used in many studies. Here, indicate whether each material, system or method listed is relevant to your study. If you are not sure if a list item applies to your research, read the appropriate section before selecting a response.

Materials & experimental systems

n/a	Involved in the study	
☒	☐	Antibodies
☒	☐	Eukaryotic cell lines
☒	☐	Palaeontology and archaeology
☒	☐	Animals and other organisms
☒	☐	Clinical data
☒	☐	Dual use research of concern
☒	☐	Plants

Methods

n/a	Involved in the study	
☒	☐	ChIP-seq
☒	☐	Flow cytometry
☒	☐	MRI-based neuroimaging

Plants

Seed stocks

N/A

Novel plant genotypes

N/A

Authentication

N/A
